# Cyclodextrin-Based Delivery Systems in Cosmeceuticals: Current Advances and Future Perspectives

**DOI:** 10.3390/biom16071011

**Published:** 2026-07-10

**Authors:** Catarina Amaro, Tomasz Kowalczyk, Laurent Picot, Anna Merecz-Sadowska, Pere Verdugo, Helena Cabral-Marques, Przemysław Sitarek

**Affiliations:** 1 Faculty of Pharmacy, University of Lisbon, 1649-003 Lisbon, Portugal; catari.amaro@gmail.com; 2 Research Institute for Medicines (iMed.ULisboa), Faculty of Pharmacy, Universidade de Lisboa, 1649-003 Lisbon, Portugal; hcmarques@ff.ulisboa.pt; 3 Students Research Group (Erasmus Student), Department of Medical Biology, Medical University of Lodz, 90-151 Lodz, Poland; 4 Department of Molecular Biotechnology and Genetics, Faculty of Biology and Environmental Protection, University of Lodz, 90-237 Lodz, Poland; tomasz.kowalczyk@biol.uni.lodz.pl; 5 UMR CNRS 7266 LIENSs, La Rochelle Université, 17042 La Rochelle, France; laurent.picot@univ-lr.fr; 6 Department of Economic and Medical Informatics, University of Lodz, 90-214 Lodz, Poland; anna.merecz-sadowska@uni.lodz.pl; 7 Eurecat, Technology Center of Catalonia, Chemical Technologies Unit, 43007 Tarragona, Spain; pere.verdugo@eurecat.org; 8 Department of Analytical and Organic Chemistry, Universitat Rovira i Virgili, 43007 Tarragona, Spain; 9 Department of Medical Biology, Medical University of Lodz, 90-151 Lodz, Poland

**Keywords:** cyclodextrins, cosmeceuticals, vitamins, UV filters, inclusion complex, drug delivery, skin penetration, solubility, topical formulation, controlled release

## Abstract

Cyclodextrins are cyclic carbohydrates capable of forming inclusion complexes with a wide range of molecules, thereby improving their solubility, stability, and bioavailability. Traditionally, cyclodextrins have been extensively applied in the food industry owing to their functionality and safety profile. However, their use in cosmeceuticals, a rapidly growing area that lies between cosmetics and pharmaceuticals, remains relatively underexplored. Given the increasing demand for scientifically validated, high-performance skincare formulations, cyclodextrins are emerging as promising compounds that can address several formulation challenges. The principal question is whether cyclodextrins represent a worthwhile investment for the future of cosmeceutical innovation. This article aims to provide a comprehensive, evidence-based overview of the current and potential roles of cyclodextrins as safe, effective, and multifunctional carriers in advanced skincare science. The current state of research on the application of cyclodextrins in cosmeceutical formulations is evaluated, with particular focus on active ingredients commonly used in dermatological care, such as vitamins A and C, coenzyme Q10, kojic acid, arbutin, and UV filters. For each of these compounds, relevant in vitro, in vivo, and clinical studies are reviewed in order to assess how cyclodextrin complexation influences key parameters, including solubility, stability, controlled release, skin penetration, and the reduction in adverse reactions. In addition, the use of cyclodextrins in the treatment of dermatological conditions such as acne, psoriasis, and rosacea is examined, highlighting their potential value, particularly in combination with azelaic acid and salicylic acid, well-known agents used to manage these conditions. Beyond their advantages, the limitations and challenges that currently restrict broader implementation of cyclodextrins in cosmeceuticals are also discussed, including cost variability, solubility constraints with certain substances, formulation incompatibilities, and regulatory considerations. Future perspectives are explored, particularly the development of novel modified and amphiphilic cyclodextrins, as well as their integration into nanotechnology-based systems and into intelligent, personalized skincare.

## 1. Introduction

The term “cosmeceuticals”, famously coined by Albert Kligman of the University of Pennsylvania in 1984, refers to a category of products that lies between cosmetics and therapeutic drugs. These products are intended to do more than simply alter appearance: they purportedly exert a therapeutic action capable of affecting the skin beyond the duration of application, although to a lesser extent than pharmaceutical drugs [1]. Kligman originally introduced this term to describe topical preparations intended exclusively for external use, whose characteristics suggested pharmaceutical activity and could offer more than mere beauty enhancement, while still not being equivalent to pharmaceutical drugs [2,3].

Even after four decades, the term remains widely used in dermatology and is broadly accepted in academic settings, as evidenced by discussions, symposia, and lectures. However, no formal recognition has been granted by regulatory bodies such as the European Union or the U.S. Food and Drug Administration. According to these authorities, a medicinal product is a substance or combination of substances used to treat, prevent, or diagnose disease, or to restore, correct, or modify physiological functions by exerting a pharmacological, immunological, or metabolic action [3]. By contrast, a cosmetic is defined as a substance or mixture intended to be placed in contact with the external parts of the human body, or with the teeth and mucous membranes of the oral cavity, exclusively or mainly for the purpose of cleansing, perfuming, changing appearance, protecting, maintaining in good condition, or correcting body odors [4]. As cosmeceuticals are considered a subclass of cosmetics, there is a difference in regulatory protocols, specifically the absence of a requirement to demonstrate safety and efficacy prior to marketing, which is otherwise mandatory for drugs [2].

A wide range of everyday products are currently classified as cosmeceuticals. Face masks, cleansing products (including lotions, powders, and shampoos), suncare products, deodorants, haircare products, perfumes, and shaving products represent only a few examples of this broad category [5]. For topical facial use, the most commonly employed ingredients include vitamins (A, C, and E), coenzyme Q10, hydroquinone, peptides, alpha- and beta-hydroxy acids, sunscreen agents, niacinamide, ceramides, zinc, and botanical extracts (e.g., licorice, aloe vera, green tea, lavender, chamomile, rose, and turmeric) [2]. Most of these ingredients are plant- or naturally derived, which, in comparison with synthetic compounds, are readily available from renewable sources (plants, seaweed, fungi, and microorganisms) and generally cause fewer adverse reactions when ingested or applied, partly because they are less efficiently absorbed. However, this also means that they are less potent and less efficient than synthetic compounds, which are designed to target specific sites and can be used at higher concentrations [6]. Consequently, advanced delivery systems are required to enhance the bioavailability and control the release of natural compounds, while also protecting them from oxidation and degradation. Moreover, as the beauty and health market increasingly favors natural products over synthetic ones, and consumers are becoming more scientifically literate and seeking environmentally sustainable options, the demand for advanced, naturally derived delivery systems continues to grow [7,8].

Cyclodextrins (CDs) have emerged as a solution to the challenges described above. They are carbohydrate-based structures obtained through the enzymatic degradation of starch, which is present in natural ingredients such as potatoes, corn, and tapioca. Owing to their architecture and high water solubility, CDs can encapsulate other molecules and form complexes, which is particularly useful for unstable or poorly water-soluble compounds [9,10]. Furthermore, they are produced by enzymatic biocatalysis, a sustainable alternative to petrochemical processes, and only negligible amounts of CDs are absorbed following oral or topical administration [11,12,13]. CDs therefore represent natural and advanced delivery systems that meet the needs of the beauty and health market, satisfy regulatory expectations, and align with consumer preferences [14].

Although the use of cyclodextrins in the broader cosmetic industry has recently been reviewed [15], a comprehensive synthesis focused specifically on cosmeceuticals, integrating in vitro, in vivo, and clinical evidence together with patents and marketed products, is still lacking. The fundamental question is whether CDs should be more extensively explored and invested within the growing field of cosmeceuticals, an area that bridges cosmetics and pharmaceuticals. Accordingly, the main aim of this work is to analyze the use of CDs in combination with some of the most relevant and frequently used active ingredients in cosmeceutical formulations, drawing on both clinical and non-clinical studies, as well as patents, inventions, and currently marketed products, in order to better understand their present status as inclusion complexing agents in cosmeceuticals. The limitations of CDs and their future potential in combination with emerging technologies are also addressed to provide a broader perspective.

## 2. Methodology

**Figure 1:**
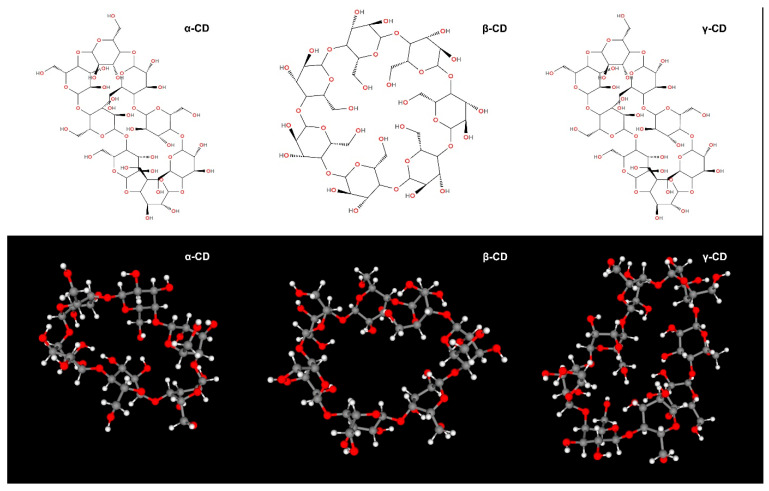
Structures of α-CD, β-CD and γ-CD shown in 2D (white background) and 3D ball-and-stick models (black background), created with MolView. In both representations, oxygen atoms are shown in red; in the 3D models, carbon atoms are grey and hydrogen atoms are white.

**Figure 2:**
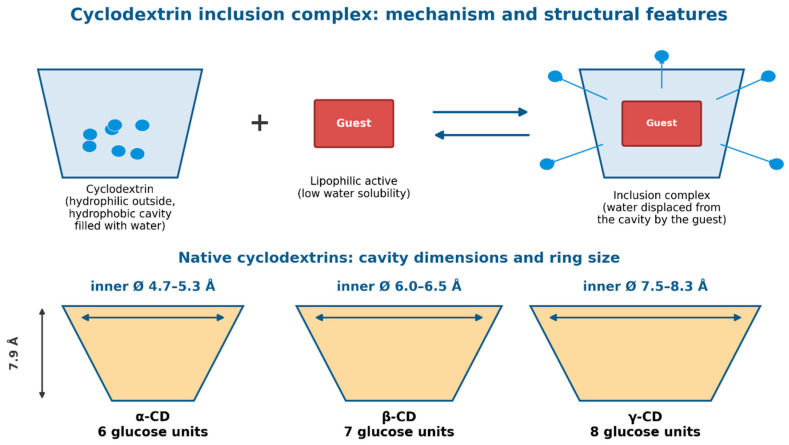
Formation and structural basis of cyclodextrin inclusion complexes. (**Top**) In aqueous solution the hydrophobic cavity of the cyclodextrin is filled with water molecules, which are displaced by a lipophilic guest to form a non-covalent inclusion complex. (**Bottom**) Truncated-cone geometry, internal cavity diameter, torus height (≈7.9 Å) and number of glucose units of the three native cyclodextrins (α-, β- and γ-CD). Figure created in Python (Matplotlib).

**Figure 3:**
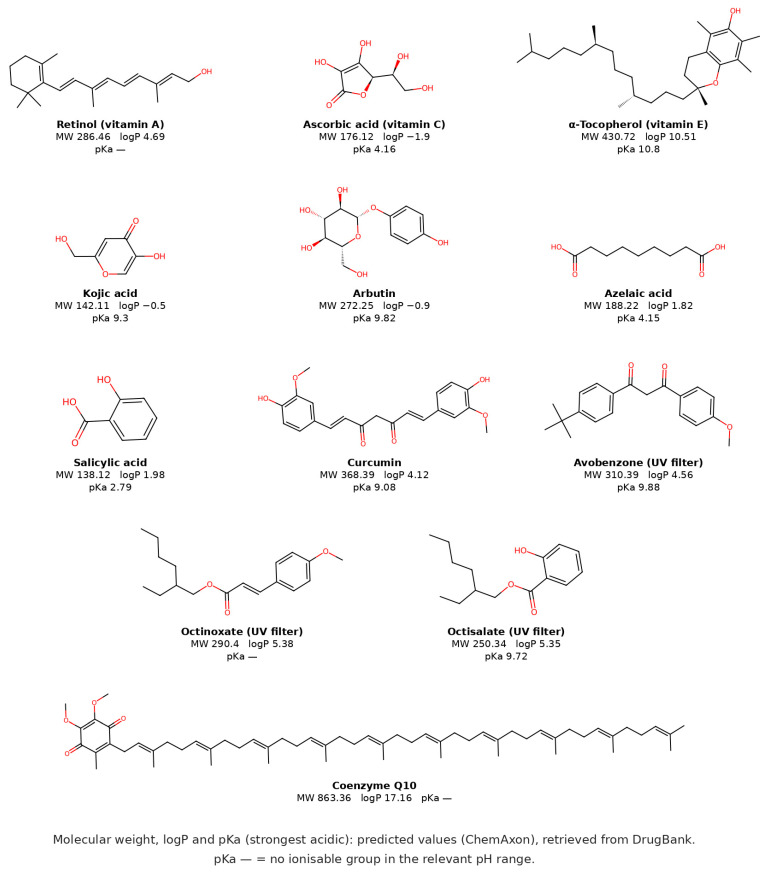
Two-dimensional chemical structures of the principal active ingredients (guest molecules) discussed in this review as candidates for cyclodextrin complexation: vitamins A, C and E, coenzyme Q10, kojic acid, arbutin, azelaic acid, salicylic acid, curcumin, and the UV filters avobenzone, octinoxate and octisalate. The 2D structures were generated by the authors in Python (RDKit) from PubChem records. For each molecule, the average molecular weight (MW), logP and strongest acidic pKa are indicated; values are predicted (ChemAxon) and were retrieved from DrugBank. A dash (—) denotes the absence of an ionizable group within the relevant pH range.

**Figure 4:**
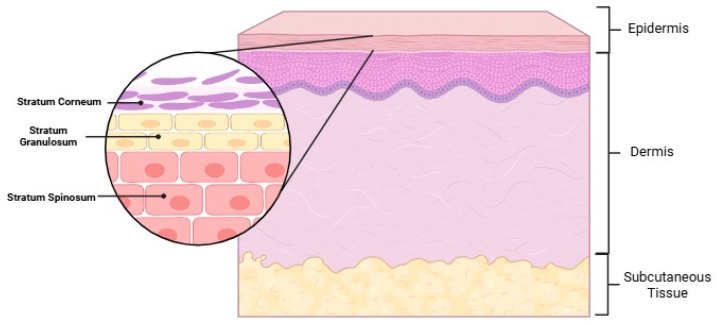
The structure of the skin is divided into 3 parts: epidermis, dermis and subcutaneous tissue. The epidermis is the outer layer of the skin and is composed of different parts, according to the type of cells. The corneocytes mentioned previously are in the stratum corneum Created in BioRender. (Amaro, C., 2025) https://app.biorender.com/profile/catarina_amaro/templates/6a4ef5cc88f19e4a1773b01f (accessed on 10 October 2025).

For the preparation of this work, a comprehensive literature review was conducted using several scientific databases, including PubMed, ScienceDirect and Google Scholar. The principal keywords used across all databases included “cyclodextrin”, “skin”, “topical”, and “cosmetic”, in combination with the names of various active substances, which were selected based on the frequency of their use in cosmeceutical products. With respect to publication dates, articles published after 2000 were preferred in order to ensure relevance and to reflect recent innovation, although earlier sources were not excluded. For articles in languages other than English, Google Translate was used to access and extract the relevant information. For the preparation of the figures, several software tools were used: MolView (Figure 1); Python with the Matplotlib library (Figure 2); Python with the RDKit library, using molecular structures retrieved from PubChem (Figure 3); and BioRender (Figure 4).

## 3. Cyclodextrins: Structure and Properties

Cyclodextrins (CDs), also known as cyclomaltoses, cycloamyloses, and Schardinger dextrins, are cyclic oligosaccharides, ring-shaped carbohydrates composed of at least six glucose units linked by α-(1,4) glycosidic bonds. They were first described in 1891 by Antoine Villiers, a French pharmacist and chemist; their cyclic structure was subsequently elucidated by Freudenberg and co-workers between the 1930s and the late 1940s, following the earlier isolation of α- and β-CD by Schardinger [14,16]. Depending on the number of glucose units present in the ring (six, seven, or eight), they are classified as α-cyclodextrin (α-CD), β-cyclodextrin (β-CD), and γ-cyclodextrin (γ-CD), respectively. Their structures are shown in Figure 1. CDs composed of rings with five or fewer glucose units cannot form due to steric hindrance, whereas those composed of nine or more units are difficult to purify [16].

The number of glucose monomers in the ring also affects the chemical properties of CDs, primarily their solubility and size. β-CD exhibits low water solubility owing to high-energy hydrogen bonding between hydroxyl groups [9], whereas the higher number of glucose monomers in γ-CD accounts for its larger structure and, in part, its high solubility [14]. All CDs share a similar non-symmetrical annular structure that extends into a truncated cone shape, with a hydrophobic interior cavity contrasting with a hydrophilic exterior [16]. This shape enables CDs to interact with other molecules, forming inclusion complexes that alter the physical, chemical, and biological properties of the guest molecules [9,16]. The mechanism of inclusion complex formation together with the cavity dimensions and ring size of the three native cyclodextrins are illustrated in Figure 2, while the main physicochemical properties of native and chemically modified cyclodextrins are summarized in Table 1. An increase in the number of glucose monomers leads to a larger structure and cavity, which can affect both the number and size of molecules that can be hosted, as well as the mode of degradation. For example, the smaller α-CD and β-CD in the crystalline hydrate state are associated with approximately 6 and 11 water molecules, respectively [17], and are resistant to salivary and pancreatic α-amylase and are instead fermented by the intestinal microbiota [14]. By contrast, γ-CD is larger than the other two and are associated with up to 17 water molecules in the crystalline hydrate, exhibiting greater flexibility, which makes it more susceptible to α-amylase, produced by the salivary glands and pancreas, and present in the duodenum [14,18]. In essence, the structure of CDs enables them to encapsulate substances, protecting them and enhancing their properties, which can be advantageous in various industries, including pharmaceuticals, agriculture, food, hygiene, and cosmetics [9,14]. Nevertheless, all CDs have limitations. For example, although γ-CD has a larger size, higher water solubility, and better bioavailability, it is more expensive than the others and is produced in lower yields, making large-scale production challenging. Consequently, α-CD and β-CD are used more frequently, despite their lower water solubility and less favorable safety profiles upon parenteral administration (β-CD, in particular, is associated with nephrotoxicity when given parenterally), and lower capacity for encapsulating guest molecules [9,19,20]. To address these shortcomings, research has led to the development of chemically modified CDs, which are obtained by introducing new functional groups into the original CDs. This approach yields new CDs with greater complexing capacity toward different substances, either through encapsulation in the cavity or through bonding at the exterior. The most common chemically modified CDs are produced by adding methyl, ethyl, hydroxyethyl, carboxymethyl, hydroxypropyl, or other functional groups, as exemplified by ethyl-γ-CD and 2-hydroxypropyl-β-CD [9,16]. In addition to chemical modification, CDs can be used to produce metal–organic frameworks (MOFs), which are highly porous structures capable of transporting or hosting active molecules within their pores or between CD–metal units, as in γ-CD-based MOFs [21,22]. CDs can also be used to produce polymeric networks cross-linked with various compounds, such as citric acid or epichlorohydrin. These materials exhibit a solid, gel-like, and porous nature, which modifies the properties of CDs. They show low water solubility and can form micro- and nanoparticles. The nature of the cross-linking agent can also affect the interaction with amylases, rendering the CDs more resistant to degradation [9,23,24,25]. With the growing use and research interest in CDs, the safety of these compounds warrants careful evaluation. According to the European Union, CDs are considered excipients with multiple functions and may be present in various preparations. With regard to dermal products, including both pharmaceutical and cosmetic formulations, CDs are poorly absorbed transdermally on their own, but are absorbed to a much greater extent under occlusive dressing conditions and/or in vehicles containing absorption-promoting agents. Concentrations of up to 0.1% of α-, β-, and γ-CD are considered safe. As for chemically modified CDs, randomly methylated β-cyclodextrin (RM-β-CD) and 2-hydroxypropyl-β-cyclodextrin (HP-β-CD) can permeate the skin by 43% and 53%, respectively, when combined with absorption-promoting agents. Furthermore, HP-β-CD is considered to be as safe as materials currently used in perfumes and cosmetics, as confirmed by various studies of antigenicity, mutagenicity, and topical irritation [13]. Thus, the parent CDs and some modified ones are safe for use in cosmetic and pharmaceutical preparations, although chemically or structurally modified CDs require additional safety assessment to characterize their risk profile.

## 4. Mechanisms of Action in Cosmeceutical Formulations

In the field of cosmeceuticals, a diverse range of components is used, the most common being, as noted above, vitamins, coenzyme Q10, sunscreen ingredients, and botanical extracts (such as chamomile and lavender), as well as plant-derived compounds, including arbutin, kojic acid, azelaic acid, and salicylic acid. The chemical structures of these active ingredients, together with their principal physicochemical parameters (molecular weight, logP and pKa), are shown in Figure 3.

These substances are used for a wide range of indications, including skin lightening or depigmentation, scar reduction, hair strengthening, anti-wrinkle and anti-aging effects, and the treatment of specific skin disorders such as melasma, acne, and rosacea [3]. While the appropriate use of these compounds can effectively address such conditions, improper use may prevent them from achieving the intended efficacy. One of the major issues with cosmetic ingredients is their limited bioavailability. As most of these compounds act on the skin, the skin itself can constitute a barrier to their effectiveness. This organ, illustrated in Figure 4, functions as a chemical, physical, and immune barrier and controls the diffusion of molecules through the outermost layer of the epidermis, known as the stratum corneum. This layer consists of corneocytes, dead, dehydrated cells arranged in a brick-like pattern in which lipids serve as the mortar. Hygroscopic molecules within the skin cells prevent water loss by attracting water [27,28]. Thus, the principal obstacle to dermal and transdermal drug delivery is the stratum corneum, as cosmetic ingredients must first penetrate this barrier to reach their site of action and exert their effect [29]. If a compound is unable to do so, its activity may be compromised by a lack of bioavailability. Examples of substances facing this issue include coenzyme Q10 and arbutin, both of which are characterized by limited skin penetration [30,31]. Conversely, certain compounds can penetrate too readily, leading to excessive concentrations that may result in adverse reactions and toxicity. A notable example is vitamin A (retinol), which, at high concentrations, can cause irritation, redness, flaking, and increased skin sensitivity [32].

Including CDs in formulations containing problematic ingredients can help to overcome these penetration and bioavailability issues. Owing to their hydrophilicity and large size, CDs do not normally penetrate the skin and therefore do not disrupt the skin barrier. By contrast, conventional penetration enhancers increase the permeability of drugs into or through the skin by temporarily altering or damaging the skin. Although the mechanism by which CDs enhance drug permeability has not been fully elucidated, it is thought to be related to the rapid equilibrium between complexed and free drug molecules, which results in rapid delivery and penetration of the drug at the skin surface without compromising the barrier. Water-soluble polymers can be added to enhance the solubilizing effect of CDs by increasing the apparent stability constants of the CD–drug inclusion complexes, without compromising the complexation capacity [29]. In addition, a study by Másson et al. concluded that the permeation-enhancing action of CDs arises from transport of the drug through the solution toward the biological membrane, where the drug molecules partition from the complex into the lipophilic membrane [33]. However, it has been reported that excessive concentrations of CDs can have the opposite effect, decreasing the penetration of lipophilic drugs through biological membranes [33,34]. The appropriate concentration of CDs in topical formulations should therefore not be overlooked, as inappropriate dosing may produce effects opposite to those intended. For compounds characterized by high skin penetration that may lead to excessive concentrations and adverse reactions, complexation with CDs is associated with improved distribution in solution. As a result, the application is more uniformly absorbed and controlled, ensuring a sustained and maximized final effect without the occurrence of side effects. This is because CDs encapsulate drug molecules within inclusion complexes and control their release through mucoadhesive properties, modulation of guest hydrophobicity, or response to external factors such as temperature, pH, and pressure. Complexation therefore protects the drug, which is then released gradually and slowly, preventing the adverse reactions that would arise from rapid release and absorption with consequent high blood concentrations. Moreover, the specific design of the CD can be advantageous: hydrophilic derivatives, such as SBE-β-CD and HP-β-CD, can enhance the solubility and bioavailability of poorly water-soluble compounds, whereas hydrophobic derivatives, such as acylated and ethylated CDs, can mediate controlled and sustained release [34]. Another issue with the cosmetic ingredients mentioned above is their tendency to degrade easily. Some compounds are highly susceptible to environmental factors, including sunlight, temperature, oxygen, and humidity. This is the case for vitamin C, which, although highly useful as an antioxidant, is very unstable and, when applied to the skin, rapidly reacts with heat, light, and oxygen [35]. Another example is vitamin E, which is more resistant to heat but remains sensitive to light and oxygen [36]. If these compounds interact with such factors, the effective concentration may not be achieved and the intended effect may be compromised. Several strategies have been developed to address this problem, including controlled production conditions, the use of amber rather than clear glass containers, and the incorporation of vacuum-pump systems, either piston-based or bag (tube)-based, into bottles [37,38]. Other approaches involve the use of preservatives or antioxidants. Although effective, these can cause adverse reactions, including skin irritation, allergic reactions, endocrine disruption, and potential carcinogenicity, as is the case with parabens [39,40]. Furthermore, such strategies address only general aspects of the formulation. By encapsulating the drug in CDs, however, the active compound is protected from all the degradation pathways mentioned above, solving the problem at its source and eliminating the likelihood of adverse reactions caused by added preservatives [9,19]. Care must nevertheless be taken in selecting preservatives, as the more hydrophilic ones may be deactivated by CDs, at least in aqueous solutions, as observed by Jansook et al. [41].

CDs can also be used to mask undesirable characteristics of compounds, particularly odor and taste. As aromas are volatile compounds, encapsulating them within the hydrophobic cavities of CDs traps them inside and prevents their release into the air, thereby reducing their volatility [42]. For instance, Hong et al. described β-CD as a potential agent for reducing the amount of sulfur-containing compounds in onions, thereby decreasing their characteristic odor [43]. However, more hydrophilic aroma compounds are less likely to be effectively isolated, as the affinity of CDs for such molecules is limited [42]. The same mechanism enables the masking of certain flavors. In addition, CDs can act synergistically with other substances to remove odorants more effectively [44]. For example, a study by Ma et al. concluded that the addition of β-CD and lactitol to goat milk significantly reduced its intense flavor and improved its sensory characteristics [45].

In summary, although the compounds most frequently used in cosmeceutical products present significant drawbacks, such as low solubility and stability, susceptibility to degradation, or sensory disadvantages, CDs can be employed to address these issues while maintaining efficacy comparable to that of alternative formulation strategies. Their potential has attracted considerable interest in various industries, including pharmaceuticals, cosmetics, and food. It is therefore essential to assess their safety, potential adverse reactions, and therapeutic effects before introducing them to the market in cosmetic and pharmaceutical preparations. Owing to the nature and mechanisms of CDs, a more detailed understanding of their interactions with the active drugs is particularly beneficial for realizing the full potential of future products [46,47]. In the following sections, the relevant research literature is presented and discussed, organized by the type of cosmeceutical formulation, the active substance with which CDs interact, and the type of study performed (in vitro, in vivo, or clinical).

## 5. Cyclodextrins in Cosmeceutical Formulations

### 5.1. Anti-Aging Formulations

One of the most popular categories of cosmeceutical products is anti-aging formulations, which aim to reduce the signs of aging, including skin wrinkles, decreased elasticity, low dermal density, and a sallow skin tone [48]. Two of the most widely used compounds in this type of cosmeceutical preparation are vitamin A and coenzyme Q10 [49,50].

#### 5.1.1. Vitamin A

Complexation with CDs has emerged as a solution to the weaknesses of vitamin A, namely its instability, whether due to UV light or temperature, and its low water solubility. This relationship has been studied and documented since at least 1983, when Pitha et al. [51] concluded that the use of CDs could increase the solubility and tissue distribution of vitamin A. Moreover, CDs alone could be used to manage hypervitaminosis A by extracting retinoids and transporting them from high-concentration tissues to organs of metabolism and excretion. In subsequent years, research has focused on enhancing encapsulation, pharmacodynamics, and pharmacokinetics, yielding formulations with fewer adverse effects and efficacy equivalent to that of vitamin A alone. More recently, preclinical and clinical studies have emerged investigating the skin penetration and therapeutic effects of vitamin A–CD complexes, which have demonstrated potential anti-aging and anti-acne properties while maintaining skin tolerance. For example, Navarro et al. [52] studied the use of β-CD and HP-β-CD to preserve the antioxidant compounds of mandarin juices enriched with pomegranate extract and goji berry juice at a 1% concentration. Although the addition of β-CD did not improve any of the evaluated parameters compared with the control juice, the juice containing HP-β-CD showed several benefits, particularly the highest values of antioxidant activity, vitamin C content, and retinol equivalents, as well as provitamin A, with a less pronounced decline over time [52].

Additional articles focusing on preclinical and clinical research are presented in Table 2, Table 3 and Table 4.

#### 5.1.2. Coenzyme Q10

Various delivery systems have been investigated to overcome the limitations of coenzyme Q10, including its instability in the presence of light, low water solubility, and high lipophilicity [69]. The complexation of coenzyme Q10 with CDs has been studied across diverse fields and has attracted considerable interest.

For instance, Nishimura et al. [70] investigated the interactions between several CDs, namely α-, β-, γ-, and HP-γ-CDs, and coenzyme Q10 in aqueous solution at a 7:1 molar ratio. Owing to the absence of precipitates in the solutions containing CoQ10 with α-CD, β-CD, or HP-γ-CD, the authors concluded that complexation occurred only with γ-CD. They described the resulting complex as a pseudorotaxane-like supramolecular structure, in which the long, lipophilic coenzyme Q10 molecule partially traverses several γ-CD rings to form a collar-like threaded assembly, rather than being partially or fully encapsulated within a single CD molecule [70].

Another study, by Naguib et al. [71], concluded that complexed CoQ10 exhibited higher stability than free ubiquinol in various aqueous ophthalmic formulations. HCEC-B4G12 human corneal endothelial cells treated with either complexed or free CoQ10 demonstrated the superiority of the complex in lowering reactive oxygen species (ROS), inhibiting lipid peroxidation, and protecting cells against erastin-induced ferroptosis, without exhibiting toxic effects. Moreover, the complex showed higher penetration and retained higher amounts of CoQ10 in human donor corneas compared with the free drug [71].

For a more comprehensive overview of the extensive studies on coenzyme Q10 and CDs, additional information is provided in Table 5, Table 6 and Table 7.

### 5.2. Skin Brightening and Depigmentation Products

Cosmetic products focused on skin pigmentation are among the most popular categories on the market. They may be used for diverse purposes, including tanning, scar treatment, and the reduction in dark spots and hyperpigmentation. By interfering with melanin production in the skin, either by inhibiting or reducing it, this type of cosmetic product promotes an even skin tone and a brighter appearance [87,88]. One of the most widely used compounds known for these actions is kojic acid, a fungal metabolite produced by several species of the genus Aspergillus. This substance inhibits the catecholase activity of tyrosinase, a key enzyme in melanin biosynthesis [89]. Although highly valued in the cosmetic industry for its depigmenting action and antioxidant properties, this compound has several limitations, namely low stability, easy degradation by light and heat, and possible adverse reactions at high concentrations, such as skin irritation and contact dermatitis [87,90]. Numerous studies have been conducted to evaluate the possibility of overcoming these limitations through complexation with CDs. For example, it has been demonstrated that CDs can reduce the penetration capacity of kojic acid, thereby decreasing its absorption into the dermis and allowing it to remain in the upper skin layers for longer to exert its whitening effect [91,92]. This effect is preserved even when sunscreen ingredients are added, which is common practice due to the photosensitivity of kojic acid, although their inclusion can significantly decrease the SPF values [91,93]. In addition, CDs can be useful for increasing the stability of W/O/W emulsions containing kojic acid, as described by Kim et al., who reported stability maintenance at 90% for 10 weeks at high temperature [94], and for slowing degradation, as they can reduce color transformation caused by light-, heat-, and oxygen-induced degradation, as reported by Hatae et al. [95]. Beyond enhancing the properties of kojic acid itself, CDs can be used to synthesize kojic acid derivatives, improved versions of the parent compound that offer advantages such as short reaction times, high yields, environmental friendliness, and simple operation under mild conditions [96,97,98]. Another common ingredient is arbutin, a hydroquinone derivative naturally obtained from various plants, including blueberries, cranberries, and bearberry leaves. It acts by the same mechanism as kojic acid, namely the inhibition of tyrosinase and the promotion of melanosome maturation [99,100]. However, its effects are more gradual and gentler on the skin, producing slower but safer results. Despite its relatively higher photochemical and acidic stability, arbutin exhibits poor water solubility, limited skin permeability, and susceptibility to hydrolysis, which can reduce its efficacy by releasing hydroquinone prematurely [101,102,103]. Furthermore, its extraction from plant tissues is challenging due to relatively low yields [104]. To overcome these drawbacks, synthetic arbutin derivatives have been developed, with α-arbutin being the most notable. Enzymes capable of producing CDs, such as cyclodextrin glucanotransferase and glycosyltransferase, are widely used in its production [104,105,106,107]. These enzymes are commonly available and relatively inexpensive. They exhibit low hydrolytic activity and high regioselectivity in their reactions, and they can act on a wide range of acceptors, yielding products in high yield [108]. Unfortunately, the direct application of CDs to arbutin has not been extensively studied: only two articles addressing complexation between these compounds were identified. The first, by Li et al., found that the inclusion complex of arbutin with HP-β-CD markedly improved the thermal stability of arbutin, with potential for expanded application in pharmaceuticals and food [101]. The second, a computational modeling and experimental evaluation by Paiboon et al., concluded that encapsulating arbutin in CDs at a 1:1 molar ratio could be advantageous, as it produced a notably slower release rate and reduced hydrolysis, without diminishing its effects [109].

In summary, although CDs are less commonly used in this type of product, their potential should not be overlooked; rather, further investment in CD-based formulations may yield improved solutions with superior performance.

### 5.3. Sunscreen and Photoprotection Products

Sunscreens and photoprotective skincare products play a crucial role in preventing undesirable dermatological consequences of chronic sun exposure, including premature skin aging and hyperpigmentation disorders. They also help prevent the development of more severe dermatological conditions, such as photodermatoses and skin cancers [110]. Most of these products rely on a combination of UV filters, which absorb, reflect, or scatter solar radiation, and antioxidant agents, such as vitamin C, which help neutralize the reactive oxygen species generated by UV exposure and provide additional protection against oxidative stress [110,111].

#### 5.3.1. UV Filters

Several studies have demonstrated the efficacy of complexing UV filters with CDs to overcome the main limitations of sunscreen agents, including transdermal permeability, photodegradation, and skin retention. For example, Scalia et al. [112] investigated the use of CDs with the UV filter 2-ethylhexyl-p-dimethylaminobenzoate (EH-DMAB). In this study, the sunscreen agent, both free and complexed with CDs, was incorporated into aqueous solutions and emulsions at a concentration of 0.15% (*w*/*w*). Several CDs were used for complexation in the aqueous solutions, namely α-CD, β-CD, HP-α-CD, HP-β-CD, and HP-γ-CD. As only HP-β-CD significantly increased the water solubility of the drug, the authors proceeded with this CD. Subsequent emulsions contained either free EH-DMAB or the drug complexed with HP-β-CD at the same concentration as in the solutions, with an additional 5% excess of HP-β-CD. The study showed that HP-β-CD significantly decreased the photodegradation of EH-DMAB in aqueous solutions, from 54.6% to 25.5%, and in emulsions, from 33.4% to 25.1%. Furthermore, complexation of the sunscreen agent with HP-β-CD limited its interaction with the skin, thereby reducing toxicity, irritation, and allergic side effects [112].

Further examples of the outcomes of combining CDs with sunscreen agents in in vitro, in vivo, and clinical assays are presented in Table 8, Table 9 and Table 10, respectively.

#### 5.3.2. Vitamin C

The relationship between CDs and vitamin C has been extensively studied across various fields. Although this compound is highly hydrophilic, it is also unstable in the presence of light, oxygen, humidity, and elevated temperatures [131]. Although the hydrophilic nature of CDs might be expected to hinder complexation, most studies report favorable results, demonstrating the adaptability of CDs. For example, Silva et al. [132] developed a β-CD-based polymer in which vitamin C is adsorbed and complexed, becoming surface-immobilized. In this system, the drug is protected from light, oxygen, and pH changes. Furthermore, the oxidation of vitamin C becomes adsorption-dependent in the β-CD-based polymer, which effectively functions as a controlled-release system. However, most studies on CD–vitamin C complexation focus on in vitro assays rather than in vivo or clinical trials, demonstrating only the potential of hypothetical cosmeceutical formulations. Consequently, limited information is available regarding marketed cosmeceutical formulations containing vitamin C–CD complexes.

Additional studies on the use of CDs with vitamin C are presented in Table 11.

### 5.4. Dermatological Cosmeceuticals (e.g., for Acne, Psoriasis)

Topical cosmeceutical preparations can include specific compounds for combating particular skin disorders. For example, salicylic acid, a compound originally derived from willow trees and other plant species [151], is useful in the treatment of psoriasis and seborrheic dermatitis, and especially of acne vulgaris, owing to its anti-inflammatory and antibacterial properties as well as its ability to dry out oily, acne-prone areas of the skin [152]. Several studies have evaluated the potential of complexation between CDs and this compound. For example, Belyakova et al. [153] developed a complexation system between salicylic acid and β-CD at a 1:1 molar ratio. The results demonstrated physical stabilization of the drug through the bonds formed between the guest and the host. Encapsulation also significantly increased thermal stability. Another study, by Căta et al. [154], also examined this CD for complexation with salicylic acid at a 1:1 molar ratio. The authors concluded that complexation occurred in both the solution and solid states, with an increase in the stability and solubility of the drug, with the latter nearly doubling in value. This may be beneficial, as larger amounts can reach the target site, leading to increased bioavailability. Furthermore, as the surface physicochemical properties of salicylic acid are altered, irritation may also be reduced [154]. In addition to these studies, patents have been issued for cosmeceutical products containing salicylic acid complexed with CDs in aqueous solution, which provides a controlled release rate of the drug at the skin surface while reducing toxic side effects [155,156]. Another example of a cosmeceutical ingredient used in dermatological formulations is azelaic acid, a compound naturally found in grains such as wheat, rye, and barley, which is beneficial for the treatment of acne vulgaris and rosacea [157]. This compound possesses antibacterial, anti-keratinizing, and anti-inflammatory properties, as well as the potential to reduce sebum production and excretion [158]. The relationship of azelaic acid with CDs has been evaluated in various systems. For example, Arpa et al. [159] studied a hydrogel composed of azelaic acid complexed with HP-β-CD at a 1.17:1 molar ratio. This system enhanced the anti-inflammatory and antimicrobial efficacy of the drug, as well as its skin penetration [159,160]. β-CD nanosponges have also been used to encapsulate azelaic acid, increasing drug solubility and providing controlled release, leading to improved safety together with adequate antimicrobial, antioxidant, and anti-tyrosinase activity. The product was obtained by suspending 10 mg of azelaic acid in 20 mg of CD nanosponges [161]. Nanovesicles containing an HP-β-CD–azelaic acid complex at a 1:1 molar ratio have also been studied; they exhibited no signs of allergy, such as erythema or edema, within 72 h on rabbit skin and showed no toxicity in mouse epidermal cell lines [162]. Manosroi et al. [163] likewise investigated inclusion complexes between azelaic acid and HP-β-CD in the solid state at a 1:1 molar ratio, which enhanced the release fluxes by approximately 41-, 81-, and 28-fold compared with uncomplexed systems through cellophane, silicone, and elastomer membranes, respectively. This difference is attributable to the increased solubility provided by encapsulation in CDs [163].

Overall, the use of CDs in dermatological and cosmeceutical formulations represents a promising strategy for enhancing both the efficacy and safety of therapeutic agents used to manage common skin disorders such as acne, psoriasis, and rosacea. Moreover, their versatility across different formulation systems, ranging from hydrogels to nanoscale vesicular carriers, demonstrates their adaptability to a variety of topical delivery platforms.

## 6. Commercial Applications and Market Examples

According to InsideCoder^®^, a website that provides descriptions of active agents and products across a wide range of areas, CDs have been extensively used in cosmetic and dermatological preparations [164]. However, in many cases, their complexation capability is not fully exploited, and they are instead employed for other purposes, such as chelating agents that inactivate impurities and heavy metals [15]. Furthermore, it is often difficult to determine the exact role of CDs in a specific product, as cosmetic formulations generally do not specify the functional role of each ingredient. When CDs are used for inclusion of the main active ingredient, however, the resulting complex itself functions as the active substance. In such cases, the formulation should ideally indicate the name of the complex formed between the active compound and the CD rather than listing the two components separately. Therefore, although formulations do not explicitly define the role of each ingredient, this does not interfere with the present analysis of cosmeceuticals containing active substances complexed with CDs, as such complexes are typically identified through distinct nomenclature for the active substance. By examining patents and technical documentation of commercial products, it is possible to identify cosmeceuticals containing CD complexes. SpecialChem, a platform that provides information on a wide variety of cosmetic and dermatological ingredients, lists several examples of such formulations. For instance, four products containing vitamin A complexed with CDs are reported [165]. Similarly, several active agents based on different concentrations of vitamin C complexed with CDs are described, most of which are intended for dermatological preparations and cosmetic products such as night masks and serums [166]. Other compounds analyzed in this work follow a similar pattern of inclusion with CDs: two products contain arbutin [167], one active agent is based on DHA, a tanning agent providing minimal UV protection [168], two products contain azelaic acid [169], and three products contain salicylic acid. These active agents are classified according to their function, such as moisturizing, repairing, anti-acne, antimicrobial, and anti-inflammatory. The advantages associated with CD complexation in these active agents and products include controlled release, increased stability, reduction or prevention of irritative reactions, and improved solubility, the last of which is particularly relevant for poorly soluble compounds such as vitamin A. However, no records were found for CDs combined with coenzyme Q10, kojic acid, or UV filters such as avobenzone, octisalate, or octinoxate [170,171,172].

## 7. Patents

With regard to patents, a considerable number address the use of CD complexes in cosmetic and dermatological preparations. In contrast to the marketed active complexes mentioned above, patents tend to focus on the potential combination of CDs with a wide range of active substances. For example, Laza-Knoerr et al. [173] describe a CD polymer and/or hydrophilic polymer emulsion containing CDs complexed with lipophilic constituents. This approach yields surfactant-free emulsions with improved stability, odor and taste masking, and enhanced solubility of otherwise poorly soluble lipophilic compounds. Another patent, by Zhang Xuanhui et al. [174], describes an oil-in-water composition containing a CD and/or its derivative, a surfactant, and a flavonol and/or its stereoisomer. Although flavonols have attracted considerable interest in the cosmetic field for their potential whitening, antioxidant, and anti-aging properties, they also suffer from limitations including poor solubility and instability in complex formulations and at high temperatures, which the proposed formulation addresses [174]. The invention by Jens et al. [175], meanwhile, focuses on the combination of CDs with quinones and their derivatives for cosmetic and dermatological skin preparations, in order to protect these compounds from oxidation and free-radical formation, thereby preventing potentially harmful reactions on the skin. Another invention, by Alexander et al. [176], further explores the synergistic combination of CDs, in this case with bioquinones and retinoids, providing a more specific application than the previously mentioned inventions. This approach offers effective protection against oxidative processes occurring either within the cosmetic formulation itself or on the skin.

Some inventions explore even more specific interactions between CDs and individual active compounds. Although these are less frequently reported than those describing broader classes of substances, they provide more detailed insights into the potential applications of such complexes. For instance, several inventions specifically address the complexation of vitamin A and its derivatives. This is the case for the patent by Jean-Christophe et al. [177], which reports the use of dimethylated β-CDs, in particular DIMEB and RAMEB, with vitamin A and its esters for cosmetic and dermatological applications. The inventors claim that the inclusion complexes formed between these CDs and vitamin A derivatives improve skin retention of the active substance while limiting its transdermal penetration. With regard to vitamin E, Regiert et al. [178] describe an invention involving a solution of α-tocopherol included in either β- or γ-CD for use in cosmetic preparations. Complexation of α-tocopherol with CDs is intended to improve the stability of this antioxidant in formulations while reducing the potential adverse reactions associated with its application. In the case of salicylic acid, He Xiaodan et al. [155] describe an aqueous solution containing a CD–salicylic acid inclusion complex, together with its preparation method and applications. The complex can be incorporated into cosmetic or medicinal products intended for contact with the skin, such as anti-acne ointments, toners, facial cleansers, and shampoos. The inventors highlight advantages such as controlled release of salicylic acid at the skin surface, ensuring an effective concentration while reducing irritant and potentially toxic side effects [155]. Similarly, a patent by Li Pengcheng et al. [156] describes a water-soluble form of salicylic acid obtained by encapsulating it in β- or γ-CDs or their derivatives. According to the inventors, this compound can be used in the preparation of cosmetic products, including anti-acne creams and toners, as well as topical medicinal formulations, which is consistent with dermocosmeceutical applications. Hyaluronic acid is associated with slightly more records than the compounds discussed above. Boiteau et al. described cross-linked hydrogels composed of CD molecules grafted onto hyaluronic acid and dextran for medical and cosmetic applications, mainly fillers, implants, and creams [179,180]. Another patent, by Jean-Guy Boiteau, describes an invention comprising hyaluronic acid and CD molecules covalently bonded to a bifunctional or polyfunctional cross-linking agent. This system is intended for medical and cosmetic applications administered dermally [181]. In both inventions, the reported advantages include controlled drug release and prolonged residence time in the human body [179,180,181]. Lastly, patents involving kojic acid complexed with CDs have also been reported. For instance, Mentink et al. describe a whitening agent composed of kojic acid encapsulated in CDs, resulting in improved stability against discoloration as well as a whitening effect [95].

Overall, the patents discussed describe the inclusion of active substances within CDs for use in cosmetic and dermatological preparations, with particular emphasis on skincare applications aimed at the treatment and management of skin conditions such as oily and dry skin, acne, and rosacea. Additional aims include the regulation of cellular metabolism and the preservation or restoration of skin condition, including improvements in skin tone, firmness, elasticity, and wrinkle depth. The available patents suggest that the use of CDs in cosmeceutical formulations for the inclusion of active substances is gradually increasing. Their advantages as encapsulating agents represent a valuable investment for improving the performance of these products. The benefits reported for the active substances described in these inventions are consistent with those discussed in the preceding sections, including reduced toxicity, improved solubility and stability, and controlled release profiles. However, the application of CDs in this field remains relatively limited, particularly with respect to their combination with other relevant active substances. For instance, their use with UV filters could be further explored, especially considering concerns regarding the systemic absorption of these agents, which CD complexation may help to limit. Furthermore, most of the existing patents focus on the most commonly used CDs. Future research exploring less frequently used CDs and their derivatives, such as macrocyclic CDs, could foster further innovation in delivery systems for cosmeceutical applications.

## 8. Limitations and Challenges

Although CDs have proven to be versatile excipients in the pharmaceutical and cosmetic fields, with promising potential as carriers for cosmeceutical ingredients, several challenges, including economic, physicochemical, formulation, and regulatory issues, continue to limit their widespread application. Understanding these limitations is essential for designing effective formulations and overcoming obstacles in product development. With regard to economic considerations, CDs are generally recognized as cost-effective ingredients, as noted above and supported by various studies. However, prices vary depending on the type of CD. For instance, in 2004 Food Standards Australia New Zealand (FSANZ) reported the costs of α-, β-, and γ-CD as approximately US$20–25/kg, US$3–4/kg, and US$80–100/kg, respectively [109]. These prices likely reflect frequency of use, as β-CD is the most widely used internationally, followed by α-CD, and then γ-CD. With the expansion of the global CDs market, a broader range of suppliers and distributors is now available. This diversification has led to significant price variability among companies. For instance, well-established chemical suppliers such as Merck/Sigma-Aldrich (Darmstadt, Germany) generally list CDs at higher prices, reflecting their focus on laboratory-grade reagents, stringent quality control, and smaller packaging volumes (typically ranging from 0.5 to 25 kg) [182]. By contrast, industrial-scale distributors such as Accio or Made-in-China, which connect buyers directly with large manufacturers, offer substantially lower unit prices, ranging, for example, from US$2 to US$10/kg for β-CD, owing to bulk purchasing options (from 10 kg up to more than 5000 kg) and reduced intermediary costs [183,184]. It should be noted that these latter figures derive from commercial supplier listings rather than peer-reviewed sources and are therefore indicative of prevailing market conditions rather than validated cost data. These differences may also be influenced by factors such as brand reputation, purity grade, packaging specifications, and transportation logistics. Thus, although the market price of CDs has generally decreased in recent years due to higher production volumes and increased competition, the final cost per kilogram can still vary considerably depending on the supply chain level, type of CD, and intended application. For chemically modified CDs and their derivatives, the production processes involve additional steps such as synthesis, purification, and drying, which require more materials, more time, and specific conditions, resulting in increased costs [185]. In cosmeceutical products, where cost efficiency is a major factor, this aspect may restrict the use of CDs to premium formulations or high-value actives. From a physicochemical perspective, the ability of CDs to improve the apparent solubility of molecules is largely restricted to lipophilic molecules and depends on structural compatibility between the guest molecule and the CD cavity [186]. This means that highly hydrophilic molecules, and those that are extremely bulky or rigid, may exhibit reduced affinity for inclusion. To overcome some of these limitations, however, CDs can be chemically modified, and larger compounds can be partially included or anchored through their hydrophilic side, or connected to multiple CDs. An additional issue is the so-called solubility inversion: CD–guest complexes can possess limited solubility and may even form precipitates, which is critical for maintaining transparency and consistency in cosmeceutical products [26]. The high hydrophilicity of the outer surface of CDs can also limit their performance in lipid-rich formulations. CDs preferentially reside in the aqueous phase, and the formation of inclusion complexes may destabilize emulsifiers or co-solvents, leading to instability in water-in-oil (W/O) systems. For example, a study by Cruz et al. showed that hydrocarbon droplets spread well over oil-wet surfaces, but that the presence of CDs reversed this effect, particularly with α-CD [187]. Therefore, when designing formulations, it is necessary to select the appropriate CD to achieve an optimal balance between complex stability and controlled release at the skin interface. In addition, research on amphiphilic CDs and polymeric CD conjugates is being developed, as these exhibit both hydrophobic and hydrophilic domains and are suitable for use in mixed or oily matrices [188]. From a regulatory perspective, CDs can present challenges. In the European Union, only certain CDs are approved as cosmetic ingredients, including β-CD, HP-β-CD, and α-CD, while others are restricted to pharmaceutical use [189]. In the United States, they are generally recognized as safe (GRAS) for oral applications, but data on their topical safety remain limited [13]. Furthermore, unlike pharmaceutical products, cosmetic products are not subject to standardized clinical efficacy testing [3]. Even when strong preclinical and clinical data are available, such as evidence of improved bioavailability or penetration due to CDs, companies are limited in the claims they can make, and terms such as “enhances dermal delivery” or “therapeutic effect” cannot be used regardless of how strong the evidence may be. Overall, while CDs offer compelling functional advantages, the diverse cost, physicochemical, and formulation limitations, together with regulatory uncertainty, continue to restrict their widespread adoption in mainstream cosmeceutical formulations. Advances in cost-effective CD production, the development of new chemically modified CDs and their derivatives, and the establishment of standardized evaluation criteria will be essential for expanding their industrial use.

## 9. Future Perspectives

As the demand for safer, more effective, and sustainable cosmetic and pharmaceutical formulations continues to grow, CDs remain an attractive platform for innovation in delivery systems. Recent advances in synthetic chemistry, the development of new materials, and the trend toward personalized skincare have expanded the potential of CDs beyond their traditional role as inclusion agents. Although α-, β-, and γ-CDs remain the most widely used, chemically modified CDs are constantly being developed to overcome the solubility, permeability, and stability limitations of the parent compounds. For example, amphiphilic CDs have been synthesized that exhibit both hydrophilic and lipophilic domains owing to the attachment of hydrophobic alkyl or acyl chains to the primary or secondary hydroxyl groups of the ring. This allows them to form micelle-like nanostructures and stabilize oil-in-water or water-in-oil systems without the need for additional surfactants, offering potential applications in water-resistant sunscreens, long-lasting foundations, and formulations combining hydrophobic and hydrophilic actives [188]. In addition, large-ring CDs, composed of at least nine glucose monomers, exhibit higher encapsulation efficiency for larger or more hydrophobic aroma molecules, such as peptides and macromolecular antioxidants [190]. However, their synthesis has so far been characterized by low yields. Advances in enzyme engineering may eventually make these macro-CDs more accessible for specialized applications [191]. As the skincare industry advances toward intelligent and personalized approaches, cosmeceuticals are also being transformed into individualized formulations. Ingredients can respond dynamically to the user’s skin condition or environment, and CDs and their derivatives, as well as materials based on them, are particularly useful for this purpose owing to their stimuli-responsive behavior and capacity for controlled release [192]. For example, a study by Ting et al. described photo-responsive CD complexes containing azobenzene derivatives that switch between α-CD and β-CD partners upon UV irradiation, resulting in slow and controlled release [193]. Another study, by Lee et al., described the fabrication of pH-sensitive CD nanoparticles containing hyaluronic acid, which enabled site-specific delivery [194]. CDs can also exhibit temperature- and enzyme-responsive behavior [195]. Such formulations could be combined with artificial intelligence, which can generate individual skin profiles, to create highly customized skincare regimens and continuously monitor changes in hydration, sensitivity, and acne patterns. It is therefore possible to deliver highly effective individualized care and to refine cosmeceutical recommendations to better match the skin’s condition and needs [196]. With regard to nanotechnology, CDs have been intensively incorporated into this field to improve the stability, solubility, and skin penetration of active ingredients. For example, they can be used to produce nanosponges, which are cross-linked CD networks with nanoscale porosity, useful for achieving sustained delivery, minimizing irritation, protecting degradable substances, and maximizing long-term efficacy [197]. When incorporated into nanoemulsions, CDs can act as both stabilizers and complexing agents, thereby reducing the need for additional compounds such as surfactants and enhancing the solubilization of lipophilic components [198]. CDs have also been used in combination with other delivery systems. Incorporating CDs into liposomes, niosomes, or nanosomes enhances drug-loading capacity and prevents the rapid release of lipophilic compounds [199,200]. When instead incorporated into microcapsules or biopolymer films, they enable better-targeted and time-controlled release, extending product longevity and efficacy [23]. An increase in research in this area, whether clinical or non-clinical, could lead to the development of additional patents, particularly involving the new technologies mentioned above and inclusion complexes consisting of active substances that are currently associated with fewer inventions, such as UV filters, plant extracts, and coenzyme Q10. As a result, more cosmeceutical products could emerge, offering various benefits and dynamic functionalities of the kind discussed throughout this work.

In summary, the continued development of novel and multifunctional CDs, combined with advances in nanotechnology and intelligent formulation design, is likely to expand their role in the cosmetic and pharmaceutical industries. New patents and corresponding products may emerge, leading to a more diverse cosmeceutical market that exploits CDs as inclusion agents.

## 10. Conclusions

CDs have proven to be remarkably versatile excipients, with growing relevance in the cosmetic and pharmaceutical fields. Although originally valued for their inclusion capabilities and solubility-enhancing properties, they are now recognized as multifunctional carriers capable of stabilizing, protecting, and modulating the release of a wide variety of active ingredients. The present work focused on the relationship between CDs and several major cosmeceutical components, with greater emphasis on vitamins A and C, sunscreen ingredients, and coenzyme Q10, and less extensive coverage of arbutin, kojic acid, and others. The aim was to analyze existing studies regarding the results obtained and the evolution of CD use with these components, with preference given to cosmeceutical applications, especially those related to the skin. As the analysis shows, the use of CDs has been extensively explored, including in the cosmeceutical field. Although research is well established for certain compounds, others remain in the early stages, underscoring the need for continued investigation focused on formulation optimization and mechanistic elucidation, since most studies have reported favorable results with high potential, highlighting the broad applicability and adaptability of these cyclic oligosaccharides. Beyond their functional versatility, CDs stand out for their safety and biocompatibility, both of which are essential for dermocosmetic applications. Their natural origin, low toxicity, cost-effectiveness, and environmental friendliness make them ideal candidates for skin-contact formulations. Acting as molecular containers, CDs can protect compounds from degradation, enhance their solubility, and minimize irritation by reducing skin penetration. All these features contribute to safer, more effective, and longer-lasting cosmeceutical products.

Although CDs are beginning to be patented as inclusion agents for cosmeceutical preparations involving a wide range of substances discussed in this work, they could be exploited further, particularly in combination with less-explored substances and newer technologies, leading to the development of novel products with characteristics and mechanisms distinct from those commonly found in the current cosmeceutical market.

The full potential of CDs in this sector will depend on the expansion of research and industrial implementation, together with the evolution of cosmetic regulations. Investment in the large-scale production of modified CDs, systematic evaluation of their interactions with biologically active compounds, and the integration of CDs into advanced delivery platforms will be beneficial for expanding their use. Strengthening collaboration between academia and the cosmetics industry will accelerate this process, ensuring that the unique advantages of CDs are incorporated into the creation of next-generation, science-driven, and consumer-safe cosmeceuticals.

## Figures and Tables

**Table 1:** Physicochemical properties of native and chemically modified cyclodextrins most relevant to cosmeceutical formulation.

Cyclodextrin	Glucose Units	Molecular Weight (g/mol)	Cavity Diameter (Å)	Water Solubility (mg/mL, 25 °C)	Degree of Substitution
α-CD (native)	6	972	4.7–5.3	145	—
β-CD (native)	7	1135	6.0–6.5	18.5	—
γ-CD (native)	8	1297	7.5–8.3	232	—
HP-β-CD	7 (β)	1541.5 a	6.0–6.5 c	>600	MS 0.6–0.9
RM-β-CD (RAMEB)	7 (β)	1303.3 a	6.0–6.5 c	>500	DS ≈ 1.8
SBE-β-CD	7 (β)	1277.1 a,d	6.0–6.5 c	>500	DS ≈ 6.5
HP-γ-CD	8 (γ)	1587.5 a	7.5–8.3 c	>500	MS ≈ 0.6

Native cyclodextrin data (molecular weight, cavity diameter, water solubility) from Saenger et al. [17]; water solubilities converted from 14.5, 1.85 and 23.2 g/100 mL; torus height ≈ 7.9 Å; cavity volumes 174, 262 and 472 Å^3^ for α-, β- and γ-CD, respectively. a Molecular weight computed by PubChem for a representative substituted structure; modified cyclodextrins are mixtures whose molecular weight, degree of substitution and solubility depend on the manufacturing grade. c For β- and γ-CD derivatives the cavity dimensions are essentially those of the parent cyclodextrin. d For SBE-β-CD, the marketed grade (Captisol) has an average degree of substitution ≈ 6.5 and a molecular weight ≈ 2163 g/mol; the PubChem value corresponds to a lower-substituted representative structure. Degrees of substitution and derivative solubilities are typical values for pharmaceutical/cosmetic grades [13,26]. MS, molar substitution; DS, degree of substitution.

**Table 2:** In vitro assays carried out with CDs and vitamin A.

Type of CDs	Type of Vitamin A	Concentration and Formulation	Cell Line	Conclusions	Ref.
β-CD, γ-CD, DIMEB and RAMEB	Propionate of vitamin A (PVA)	Skin penetration studies: Aqueous gel containing Retinol (from 0.45 to 0.96% *w*/*w*) and RAMEB (from 25 to 53.3% *w*/*w*) For stability studies: Aqueous solutions with either 100 mL of 100 mM βCD + 10 mM PVA or 20 mL of 50 mM γCD + 5 mM PVA	Fresh human skin pieces	B-CD accelerated PVA degradation. Inclusion in γ-CD protected it for at least 6 months, even if in presence of light or oxygen, although it decreases skin penetration. RAMEB could make a very stable and soluble complex with PVA, enhancing its penetration in the skin.	[53]
HP-β-CD and β-CD	Retinoic acid	Solid Dispersions made with physical mixing or kneading techniques.	hFB-4 Human fibroblast cells	CDs increased retinol solubility (less than 1 μg/mL) between 5.5 and 32.5 μg/mL, depending on the CDs and preparation techniques used. The kneading technique had better results regarding solubility, showing it was a better method for dispersion preparation	[54]
E-γ-CD, O-γ-CD	All-trans-retinol	CDs/retinol mixtures in water/ethanol (95/5 *v*/*v*)	Ear porcine skin	Alkyl-CDs increased Retinol solubility, stability and approximately 1.5-fold retinol skin accumulation and absorption, thus improving its effectiveness. The retinol did not reach the circulation.	[55]
Dimethyl-β-CD	All-trans retinoic acid	0.05% tretinoin hydrogel	Fresh Pig Ears	The complexation increased the overall tretinoin diffusion by enhancing its solubility. Retinol skin retention also increased from 0.17 ± 0.04% to 0.41 ± 0.08%.	[56]
HP-β-CD	Isotretinoin	Isotretinoin–CD complex in solution and in loaded elastic liposomal	Excised albino abdomen rat skin	Complexation of isotretinoin with HP-β-CD increased its skin deposition significantly, and by more than 21-fold if in elastic liposomes, than the Isotretinoin solution. Photodegradation also drastically slowed from 52.2% to 7.8% after 1 h of UV light. Combining complexation with elastic liposomes, the photodegradation went from 98.5% to 27.9% after 6 h.	[57]
β-CD and EPI-β-CD	Tretinoin	Hydrogels made with Tretinoin-EPI-β-CD loaded nanostructured lipid carriers’ suspensions (93%, *w*/*w*)	Goat skin	EPI-β-CD exhibited greater solubilizing properties than β-CD. The simultaneous presence of nanostructured lipid carriers (NLC) and EPI-β-CD improved permeation rate (increased >1.4 after 8 h compared to a gel with only Tretinoin) and dissolution. The synergistic effect of CDs and NLC brings advantages for therapy efficiency.	[58]
HP-β-CD	All-trans-retinoic acid	HP-β-CD-based all-trans-retinoic acid solution	Not applicable	Complexation of all-trans-retinoic acid with HP-β-CD improved its photostability and slowed photodegradation rate.	[59]
RAMEB and Chol-βCD-Ac	Vitamin A propionate (PVRA)	Gel with PVRA-Chol-βCD-Ac loaded nano capsules in aqueous suspensions or PVRA-Rameb aqueous solutions.	Fresh human skin pieces	Colloidal suspension can be used to form the nano capsules gel which allows good penetration in the skin, reproducible size distribution and long-term stability.	[60]
Amphiphilic-β-CDs: S-β-CD and O-β-CD	All-trans-retinol	Nano-vesicle solutions of all-trans-retinol with either S-β-CD or O-β-CD.	Not applicable	The CDs gave high stability to the labile compound against UV irradiation, particularly the S-β-CD.	[61]
γ-CD-MOFs	VAP	γ-CD-MOFs/VAP solutions and solid particles	Not applicable	Complexation of VAP in γ-CD-MOFs improved its stability, up to more than 1.6-fold elongated half-life, being comparable or even better to the best available reference product in the market, which was Vitamin A powder from BASF, a leading global supplier of stabilized vitamin A.	[62]
HP-β-CD-NW and HP-γ-CD-NW	Vitamin A acetate	Nanofibrous webs of CDs and Vitamin A acetate/CDs (solids and solutions)	Not applicable	The nanofibrous webs with Vitamin A acetate-CDs improved antioxidant power, solubility and thermal stability of Vitamin A acetate, with the HP-β-CD-NW showing better results and potential.	[63]
β-CDs	VAP	Inclusion complexes of Vitamin A Palmitate with β-CDs in pellets.	Not applicable	There was a notable increase in Vitamin A Palmitate water solubility and stability against temperature, oxygen and UV light. The complex could be used in emulsion formulations.	[64]

**Table 3:** In vivo assays carried out with CDs and vitamin A.

Type of CDs	Type of Vitamin A	Concentration and Formulation	Animals	Via, Dose, Duration	Mechanisms and Effects	Ref.
HP-β-CD	Isotretinoin	Isotretinoin–CD complex solution	18 Male albino rabbit	Topical, 0.5 mL every 24 h. 3 days	The isotretinoin–CD solution had significantly less irritation showing (less than half of the isotretinoin solution erythema score). If in an elastic liposomal formulation, the Isotretinoin–CD complexes had even lower scores. Thus, vitamin A with CD is less irritating than the free drug, and even more secure if the complex is converted into liposomes.	[57]
HP-β-CD	Isotretinoin	Isotretinoin–CD complex solution and elastic liposomal formulation with isotretinoin–CD	15 Sprague Dawley rats	Topical, once. 1 day.	No damage was observed in the stratum corneum when isotretinoin is complexed to CDs, in solution or in elastic liposomes, contrary to the free drug which showed partial damage.	[57]

**Table 4:** Clinical assays carried out with CDs and vitamin A.

Type of CDs	Type of Vitamin A	N	Concentration and Formulation	Application and Dosage	Exposure Time	Conclusions	Ref.
HP-β-CD	All-trans-retinoic acid	12 photoaged women and 3 men	RA/CD 0.05% in a moisturizing base	Topical, one side of the face, twice a day	8 weeks	Retinol rejuvenation effect was retained by complexation with HP-β-CD, which also improves photostability and solubility and adverse reactions, including irritation, erythema and inflammation. The effects were recorded at a histological level.	[65]
HP-β-CD	All-trans retinoic acid	12 female patients	RA/HP-β-CD 0.1 wt% in a moisturizing base	Topical, at the lateral angle of the eye. One side of the face.	8 weeks	The wrinkle areas decreased more than 20% within 8 weeks. No skin irritation was reported. Erythema, dryness and skin desquamation were less with the complex than with vitamin A only.	[66]
HP-γ-CD	Retinol and derivatives	32 healthy female and male patients	Essence made with supramolecular/capsulated retinol in CDs. Occlusive patch test: 20 µM 8 weeks clinical study: a small amount every other day at first and increase to twice daily after	0.05%, 0.1%, 0.2%, 0.3% and 0.5% of supramolecular retinol (for occlusive patch test) and 0.1% for the 8 weeks clinical study.	24 h (for the occlusive patch test) and 8 weeks (for clinical study)	Regarding skin irritation, the retinol-CDs essence was more tolerant than the non-capsulated one, with approximately half of the doubtful reactions, although they increase with higher concentrations. 0.1% is the optimal concentration for the Asian population. In the 8 weeks clinical study, the 0.1% retinol-CDs essence showed to be an effective and gentle approach to various photoaging issues for Chinese facial skin.	[67]
β-CD	Retinoic acid (RA)	66 male and female patients with acne vulgaris	RA/β-CD in hydrogel and moisturizing base	0.025% of RA/β-CD. Nightly topical application	3 months	The topical RA-CDs, either in base or gel, were more effective than the twice concentrated commercial RA comparator. RA-CDs in a moisturizing base was the best formulation in the study, with no adverse reactions and offered better skin hydration. RA-CD complex is an effective and well-tolerated alternative for the treatment of acne vulgaris.	[68]

**Table 5:** In vitro assays carried out with CDs and CoQ10.

Type of CDs	Concentration and Formulation	Cell Line	Conclusions	Ref.
γ-CD-MOFs	Physical mixture containing either 1:1 or 1:2 of CoQ10 and γ-CD-MOFs. A concentrated sample prepared by solvent evaporation, with a molar concentration of 1:1 and 1:2 of CoQ10-γ-CD-MOFs.	Not applicable	Complexation occurred at a molar ratio of 1:2 of CoQ10-γ-CD-MOFs. The sample prepared by solvent evaporation at this concentration had better dissolution in water. γ-CD-MOFs can be used as a new carrier for CoQ10.	[72]
γ-CD	Hydrophilic ointments containing solid CoQ10-γ-CD complexes prepared by: kneading method with a molar ratio of 1:5 of CoQ10 and γ-CD; solubility method with 44.2 mg of CoQ10 and 2 mL of aqueous γ-CD (232 mg/mL), with an ending CoQ10-γ-CD molar ratio of 1:7. Both kneading and solubility methods had a heated and non-heated variant, where preparation and incubation occurred at 50 °C, respectively. A physical mixture of 1:5 CoQ10-γ-CD was also prepared.	Not applicable	Complexation of CoQ10 with γ-CD occurred in the kneading and solubility method, with heating improving the results. The solid complexes obtained from solubility plus heating method improved the release rate of CoQ10 from hydrophilic ointment, thus those complexes can be used in ointments.	[73]
γ-CD	Samples containing 60 mg of CoQ10-γ-CD in 3 mL of Milli-Q water, with or without 33 mg of sodium taurocholate (Na TCA).	“LabCyte EPI-MODEL” human three-dimensional cultured epidermal model	When the complex interacts with Na TCA, CoQ10 is substituted by it, forming Na TCA-γ-CD complexes. The dissociated CoQ10 is surrounded by surface-active Na TCA, leading to a nanometer size molecular captured micelles. The water solubility and bioavailability are greatly enhanced, making it also useful for epidermis permeability.	[74]
3 polyamine-modified β-CD (host 1, 2 and 3)	Complexes prepared by suspension method: excess amounts of CoQ10 (150 mg) were added to 2 mL of water and increasing amounts of polyamine-modified β-CDs (0–0.274 M). The undissolved CoQ10 was filtered, resulting in a solution with only CoQ10-polyamine-modified β-CDs.	Not applicable	Solubility of CoQ10 increased linearly when added to all 3 types of CDs. The host 2 showed the highest values (1.52 mg/mL compared to 1.35 and 1.44 mg/mL from host 1 and 3, respectively) and was the most suitable for complexation with CoQ10.	[75]
γ-CD	Aqueous samples containing a 0.4 molar ratio of CoQ10 to γ-CD, in addition to dipotassium glycyrrhizate (GZK2).	Not applicable	GZK2 acted as a guest compound of γ-CD in the complex CoQ10-γ-CD, forming CoQ10 micelles with high solubility.	[76]
β-CD and γ-CD	Aqueous solutions containing CoQ10 and β-CD (with concentrations 0.0867 M and 0.088 M, respectively) or CoQ10 and γ-CD (with concentrations 0.38 M and 0.39 M, respectively)	Not applicable	Solubility of CoQ10 in presence of CDs increased linearly with temperature and pH. Complexation strongly enhanced Photo and thermal stability, with more than 64% of CoQ10 unchanged after 2 h exposure in 80 °C and UV light.	[77]

**Table 6:** In vivo assays carried out with CDs and CoQ10.

Type of CDs	Concentration and Formulation	Animals	Via, Dose, Duration	Mechanisms and Effects	Ref.
Not specified (Product bought as CoQ10-CD)	Water-soluble powder	Male Sprague-Dawley rats (*n* = 240)	Single oral dose of CoQ10-CD, 60 mg/kg body weight, 24 h.	The powder showed high solubility and dispersibility in water. Plasma levels of CoQ10 in rats tended to be higher after CoQ10-CD administration in comparison with crystalline CoQ10, showing a 20% higher relative bioavailability compared with the crystalline CoQ10.	[78]
α-CD, β-CD, γ-CD and HP-β-CD	Gelatin capsules containing solid complexes of CD-CoQ10 prepared by the kneading method, at 1:1, 1:3, 1:5 and 1:10 molar ratios of CoQ10/CDs.	Male beagle dogs	Oral, equivalent to 30 mg of CoQ10 (CDs’ concentrations from 0 to 100 mM/L), 1 h.	Complexation with γ-CD increased the solubility, oral bioavailability and dissolution rate of CoQ10. Dissolution rate also moderately increased when CoQ10 complexed with β-CD, whereas α-CD and HP-β-CD only showed a small increase.	[79]
HP-β-CD	Water-soluble CoQ10 serum with 2% CoQ10-HP-β-CD, obtained by trituration.	Healthy male Wistar rats (*n* = 2)	Topical, applied over 6 cm^2^ of skin, 24 h.	The serum shows a shear-thinning behavior, enhancing its application and performance. In addition to stability longer than 3 months, the serum had a high-water solubility (17.5 ± 1.8 mg/mL), a great encapsulation efficiency (retained 71% of CoQ10) and an optimal pH of 4.3, enhancing skin compatibility. During the 24 h, the rats showed no signs of irritation, or redness, suggesting its potential safety for application. This formulation seems favorable for skincare products, although the very small sample size (*n* = 2) limits the strength of this conclusion.	[80]
β-CD	Powder containing 26% *w*/*w* of CoQ10 in the form CoQ10-β-CD. It was diluted in water before administration.	Thoroughbreds horses (*n* = 19, 11 males, 8 females)	Oral, daily, 1.5 mg/kg body weight of CoQ10-β-CD, 9 weeks.	Plasma concentrations of CoQ10 and mean skeletal muscle complex I + III activity increased significantly in all horses.	[81]

**Table 7:** Clinical assays carried out with CDs and CoQ10.

Type of CDs	N	Product	Application	Exposure Time	Conclusions	Ref.
γ-CD	35 Japanese volunteers (30–60 years) consuming cigarettes daily (10–20 cigarettes/day)	Dark brown capsules containing CoQ10γ-CD complex.	Oral, 3 times a day (equivalent to 30 mg of CoQ10γ-CD/day	6 weeks	Complexation enhanced bioavailability, achieving long-lasting high plasma CoQ10 levels. After 6 weeks, wrinkles were decreased visually while skin elasticity increased. Urinary 8-OHdG, suggesting a reduction in risk of cancer. A decrease in CPK and LDH levels also suggest CoQ10 acts as a radical scavenger with antioxidant effects, protecting muscle cells.	[82]
γ-CD	22 healthy adults (12 males and 10 females)	Capsules containing CoQ10-γ-CD complex	Oral, single dose, 30 mg of CoQ10-γ-CD	2 weeks	The single-dose of CoQ10-γ-CD increased 47.6% plasma CoQ10 levels, with higher maximum concentration and AUC values, in comparison to CoQ10 with microcrystalline cellulose, which only had a rise of 13.83%. Complexation with γ-CD can significantly increase oral absorption and bioavailability of CoQ10.	[83]
β-CD	5 subjects (2 women, 3 men) aged from 24 to 50 years.	Hard gelatin capsules containing CoQ10-β-CD	Oral, single dose, equivalent to 180 mg of CoQ10	24 h	The CoQ10-β-CD complex in the capsules had a sustained release, with its bioavailability significantly better than the crystalline CoQ10.	[84]
β-CD	22 subjects, aged from 25 to 57 years.	Hard gelatin capsules containing CoQ10-β-CD	Oral, equivalent to 60 mg of CoQ10/day	21 days	The complex CoQ10-β-CD’s sustained release and higher uniform bioavailability were confirmed. All subjects that took CoQ10-β-CD capsules had at least a doubling in the plasma CoQ10 levels after 21 days (representing a 100% response rate, whereas the solubilized CoQ10 had only 44%).	[84]
β-CD	14 healthy non-smoking male volunteers (aged from 30 to 52)	Water suspension and powder containing 7.5% and 5% of CoQ10/β-CD, respectively.	Oral, single-dose (one formulation each week), equivalent to 60 mg of CoQ10	3 weeks	Complexation of CoQ10 with β-CD enhanced water solubility, oral absorption and bioavailability, with the last one having potential to further improve.	[85]
α-CD, β-CD, γ-CD and β-Iso (a mix of the other CDs and their maltosyl maltose version)	20 healthy female volunteers	Samples containing either CoQ10-β-CD, CoQ10-γ-CD or CoQ10-β-Iso. Samples were administered with water.	Oral, single dose, equivalent to 0.30 g of CoQ10.	24 h	Complexation with α-CD did not occur, whereas it did with the others. β-CD, γ-CD and β-Iso improved and accelerated the absorption of CoQ10.	[86]

**Table 8:** In vitro assays carried out with CDs and sunscreen blockers.

Type of CDs	Sunscreen Agent	Concentration and Formulation	Cell Line	Conclusions	Ref.
β-CD	Avobenzone	Solution made with 50 µM of Avobenzone and β-CD (ranging from 0 to 10 mM)	Not applicable	Aggregation with CDs enhanced Avobenzone’s photostability and, at concentrations more than 2 mM, increased UVC band absorption.	[113]
β-CD	Avobenzone, Oxybenzone and Ensulizole	Creams as oil/water emulsions (1%*w*/*w* of β-CD)	Abdominal skin of Wistar male rats	Complexation with β-CD reduced skin absorption after 6 h of the UV filters 6 to 17-fold while also prolonging their lag time of absorption to more than 150 min.	[114]
Mono-chloro triazinyl-β-CD	Octinoxate	Tencel fabric treated with CD solution and a solution with the sunscreen agent.	Not applicable	The fabric treated with CDs had the uptake of sunscreen increased by 3.2-fold compared to untreated one, exhibiting also higher photo protective capacity.	[115]
pγCD	Avobenzone and Octinoxate	Solutions containing one of the sunscreen agents and pγCD.	Not applicable	Formation of inclusion complexes with pγCD highly increased the water solubility for both sunscreen agents and the photostability of Avobenzone, although accelerated the photodegradation of Octinoxate.	[116]
HP-β-CD	Avobenzone	Aqueous formulations of 0.12 mg/mL Avobenzone and 0, 10, 20 or 30% (*w*/*w*) of HP-β-CD. If less than 20% *w*/*w*, it’s considered a suspension. If concentration is 20 or 30% *w*/*w*, the formulation is a solution)	Hairless mouse skin from 6-week-old female SKH-1 mice with no hair	HPCD increased Avobenzone’s solubility significantly, from 21.7 µg/mL to 375.0 µg/mL in the highest CD concentration. Similarly occurred to Avobenzone’s transdermal permeability and flux, with the formulation 20% HP-β-CD achieving the best results in both. HP-β-CD at higher concentrations, especially 30%, greatly suppresses photodecomposition, preserving almost double of avobenzone when compared with the drug alone.	[117]
HP-β-CD	Oxybenzone	Aqueous formulations of 2.67 mg/mL Oxybenzone with HP-β-CD varying from 0, 5, 10 and 20% (*v*/*v*). The formulation is a solution if HP-β-CD is 10 or 20%, and a suspension otherwise.	Skin samples of 6-week-old male SKH-1 hairless mice	HP-β-CD significantly increased the aqueous solubility, transdermal permeation and flux of the drug, with the 10% HP-β-CD formulation showing the best results. Similarly, was registered for skin accumulation of the drug with the 5 and 10% HPCD formulations exhibiting the highest accumulation values.	[118]
HP-β-CD	Oxybenzone Octocrylene, and Octinoxate	Aqueous solutions made of different sunscreens and HP-β-CD in different molar ratios (0:1, 1:1, and 2:1). Cream formulations (oil-in-water emulsions) containing sunscreen lotions alone (2% *w*/*w*) or complexed with HP-β-CD (0, 5 or 10 g).	Not applicable	Complexation with CDs increased photostability of Octinoxate and Octocrylene, with the last one up to six- to eightfold. Regarding resistance against dehydration, loss in lotion’s weight decreased as amounts of HP-β-CD increased.	[119]
HP-β-CD and SBE-β-CD	Avobenzone	Solutions containing avobenzone free or complexed with CDs (1:2 molar ratio of Avobenzone to HP-β-CD or SBE-β-CD), in concentration 15 µg/mL	Excised human female skin tissues (breast and abdomen)	Complexation of Avobenzone with SBE-β-CD leads to high sunscreen levels at the skin surface with reduced retention in the epidermis, reducing potential photo-damage to bio substrates.	[120]
β-CD	Oxybenzone, octocrylene and Octinoxate	Aqueous solutions made of different sunscreens and CDs in different molar ratios (0:1, 1:1, and 2:1). Cream formulations (oil-in-water emulsions) containing sunscreen lotions alone (2% *w*/*w*) or complexed with β-CD (0, 5 and 10 g).	Not applicable	Adding β-CD in either CD concentration increased the photostability of cream formulations as it slowed down photodegradation. Regarding resistance against dehydration, the loss in lotion’s weight decreased with increasing amounts of β-CD.	[121]
2-HP-β-CD	Avobenzone	Aqueous solutions derived from 2 mmol of 2-HP-β-CD and 1 mmol of Avobenzone (0–15 mmol/L of CDs). Solid powder samples resulted from the solution’s lyophilization.	Not applicable	The complexation with 2-HP-β-CD increased water solubility, thermal and photostability of avobenzone.	[122]
β-CD, HP-β-CD, HP-α-CD and HP-γ-CD	Trans-EHMC	Methanolic solutions containing CD-drug complex in equivalent amounts of trans-EHMC (0.18%, *w*/*w*). Lotion formulations (oil-in-water emulsion) with trans-EHMC alone or complexed with CDs (0.2%, *w*/*w*).	Not applicable	Only β-CD and HP-β-CD showed results. Both reduced the drug’s decomposition in alkaline solutions. HP-β-CD was more effective and reduced irradiation induced degradation. The complexation with β-CD improved the chemical and photo-stability of the sunscreen agent.	[123]
α-CD, β-CD, HP-α-CD, HP-β-CD and HP-γ-CD	BM-DBM	Solutions and lotion formulations (oil-in-water emulsion), both containing free or complexed BM-DBM (both 0.15% (*w*/*w*)).	Not applicable	β-CD and HP-B-CD produced the best results in increasing the water solubility of BM-DBM, but only HP-β-CD produced a remarkable increase. This CD also improved photostability by reducing loss of sunscreen agent from photodegradation (70.4 to 49.2%).	[124]
HP-α-CD, HP-β-CD, HP-γ-CD and RM-β-CD	Phenylbenzimidazole sulfonic acid (PBSA)	Aqueous solutions with 1:1 and 1:10 molar ratios of PBSA to CDs, depending on preparation method (kneading and co-evaporation). Cream formulations (oil-in-water emulsion) with the free sunscreen or complexed with CDs (both cases with (0.5% *w*/*w*)).	Not applicable	Both HP-β-CD and RM-β-CD had the best results in increasing water solubility of PBSA. HP-β-CD also significantly reduced irradiation-induced decomposition of the drug (from 9.1 to 3.9%) and suppressed its photosensitizing potential by almost completely inhibiting the drug’s production of free-radicals. Regarding long-term stability tests (6 months, room temperature, in the dark), the HP-β-CD formulation retained 99.1% of the initial sunscreen concentration, while the uncomplexed one fell below 81.0% of the original concentration.	[125]
p-β-CD in the form of p-β-CDE and p-β-CDC	4-tert-butyl-4′-methoxydibenzoylmethane (DBM) and 2-ethylhexyl-p-meth oxycinnamate (EHMC)	Aqueous solutions containing excess of sunscreen agent and CDs.	Not applicable	Complexation of the compounds increased significantly water solubility for each sunscreen agent. EHMC had the highest amount when complexed either with pbCDE or pbCDC, with the last CD having the lowest amounts of either sunscreen agent. The photostability of DBM and EHMC increases when complexed with pbCDE while pbCDC decreases DBM photostability.	[126]
β-CD	Octinoxate	Gel creams containing either only 13.26 g of β-CD-OMC or 6.63 g of β-CD-OMC plus 25 mL of OMC’s liposomes	Skins from the ears of 4-month-old female pigs	Complexation with β-CD increased permeation of the drug from the epidermis to the dermis, which can be a disadvantage. The formulation with β-CD and OMC’s liposomes had better results, possibly due to liposomes compensating over CDs.	[112]

**Table 9:** In vivo assays carried out with CDs and sunscreen blockers.

Type of CDs	Sunscreen Agent	Concentration and Formulation	Animals	Via, Dose, Duration	Mechanisms and Effects	Ref.
HP-β-CD	Avobenzone	Aqueous formulations of 0.12 mg/mL Avobenzone and 0, 10, 20 or 30% (*w*/*w*) of HP-β-CD and (if less than 20% *w*/*w*, it’s considered a suspension, otherwise it’s a solution)	SKH-1 hairless mice (*n* = 4)	Topical, 2 mg/cm^2^, 24 h	All formulations containing HP-β-CD reduced significantly skin edema and sunburn cells. The 30% HP-β-CD formulation had the best results with nearly no edema and the lowest levels of sunburn cell.	[117]
HP-β-CD	Oxybenzone	Aqueous formulations containing 2.7 mg/mL oxybenzone and up to 20% (*w*/*w*) HP-β-CD. Considered solutions if 0 or 5% HP-β-CD and suspensions if 10 or 20% HP-β-CD.	SKH-1 hairless mice (*n* = 4)	Topical, 2 mg/cm^2^, 24 h	Complexation with CDs enhanced the photoprotective effects of the sunscreen agent, without preventing it from interacting with light. The 5 and 20% HP-β-CD formulation showed significantly less edema, with the last formulation also exhibiting the lowest count of sunburn cells.	[127]
HP-β-CD	Benzophenone-3	Sunscreen formulations with 4% BZ-3, with or without complexated with HPCD in stoichiometric ratios of 1:1 and 1:2.	Adult male albino New Zealand rabbits (*n* = 18)	Topical, daily for 15 days	CD significantly increased drugs aqueous solubility and reduced systemic penetration. The formulation with this complex showed no comedogenic potential, contrary to free UV filter formulation alone.	[128]

**Table 10:** Clinical assays carried out with CDs and sunscreen blockers.

Type of CDs	Sunscreen Agent	N	Product	Application and Dosage	Exposure Time	Conclusions	Ref.
HP-β-CD	Avobenzone	6 healthy Caucasian volunteers, aged 20–50 years, without dermatological disorders	Cream (oil-in-water emulsion)	Topical on the forearm, 2 mg/cm^2^ in an area of 2 cm × 5 cm	60 min	Complexation with CDs increased water solubility of the drug and accumulation in outer layers of the skin, enhancing its protective power. It also reduces drug penetration in inner layers, therefore limiting potential toxic reactions.	[129]
HP-α-CD, HP-β-CD, HP-γ-CD and RM-β-CD	Enzacamene	6 female volunteers, aged 25–30 years and without dermatological disorders	Cream (oil-in-water emulsion) containing Enzacamene (1.0%, *w*/*w*) alone or complexed with CDs (both cases 1.0%, *w*/*w*, 1:1 molar ratio)	Topical on the intern forearm, 2 mg/cm^2^ in an area of 2 cm × 5 cm	30 min	RM-β-CD showed better potential in increasing the water solubility and reducing the photodegradation of Enzacamene.	[130]
β-CD	Octinoxate	10 volunteers with skin phototypes (SPT) I, II, and III, aged 18–42 years	Gel creams containing either only 13.26 g of β-CD-OMC or 6.63 g of β-CD-OMC plus 25 mL of OMC’s liposomes	Topical on the backside, 2 mg/cm^2^	3 days	Inclusion with β-CD did not increase the SPF value, nor water resistance.	[112]

**Table 11:** In vitro assays carried out with CDs and vitamin C.

Type of CDs	Form of Vitamin C	Concentration and Formulation	Conclusions	Ref.
2-HP-β-CD	Ascorbic acid (AA)	Aqueous solutions containing increasing concentrations of CDs (0, 125, 375, 750 and 1000 µM) and ascorbic acid extracted from fresh kiwi (6 µM) or commercial bought (6.8 µM)	The CDs acted as a secondary antioxidant, enhancing the antioxidant capacity of the system.	[133]
α-CD, β-CD	AA	Fresh or aged aqueous solutions containing ascorbic acid and increasing concentrations of CDs (from 1.3 to 10 mM).	Complexation with β-CD increased stability of the drug, making its oxidation difficult while not affecting its behavior. α-CD did not show relevant results.	[134]
HP-β-CD	L-Ascorbic acid (LAA)	Clear transparent solutions containing ranging concentrations of LAA-HP-β-CD in a 2:1 molar ratio: 10–30 µM of LAA and 5–15 µM of CDs.	Complexation increased the thermal degradation temperature of LAA, therefore enhancing its stability. In addition, LAA-HP-β-CD had a higher antioxidant potential than LAA alone.	[135]
γ-CD	AA	Aqueous solutions containing either complexes of AA-γ-CD, with 0.05 mM of AA and 0–100 mM of γ-CD, or complexes of AA-γ-CD-Poly(vinyl Alcohol), with 0.02 mM of AA, 5–10 mM of γ-CD and 100–400 of Poly(vinyl Alcohol)	Complexation with γ-CD increased significantly inhibition of AA, therefore protecting it from oxidation reactions, either in binary or ternary complexes.	[136]
γ-CD	VC-IP	White powders produced from ethanolic solutions containing 459 mg of VC-IP and 1 g of γ-CD.	γ-CD leads to faster drug release since the complex showed significantly higher release values after 5 min and 7 h, compared to the pure active compound.	[137]
β-CD	AA	Polyvinyl alcohol nanofiber webs containing β-CD-AA inclusion complexes (17%, 25%, and 33% (*w*/*w*) of β-CD and equimolar concentrations of AA)	PVA nanofiber webs containing β-CD-AA inclusion complexes were successfully produced and could be the first step using β-CD-AA inclusion complexes for cosmetic and topical drug delivery applications.	[138]
β-CD	AA	Solutions containing 0.1 mM of AA and different concentrations of β-CD (0, 0.05, 0.1, 0.15, 0.2 mM)	Complexation of AA with CDs was successfully achieved, being more stable than the dopamine-CD complex. In addition, complexation makes the ascorbic acid more stable and protects it from oxidation.	[139]
β-CD	L-AA	PVA nanofibers electrosprayed with solutions containing 5% (*w*/*v*) of LAA-loaded β-CD (1:1 molar ratio)	This delivery system can protect Vitamin C, increase its heat resistance while release it constantly with a zero-order release profile, important for therapeutic action. This system could be incorporated into a skin patch.	[140]
β-CD	AA	Aqueous solutions (1:1 molar ratio)	Complexation between AA and β-CD is thermodynamically favorable, with a higher order of association than the complexation of nicotinic acid with β-CD.	[141]
α-CD, β-CD	AA	Aqueous solutions containing 1.10 ± 0.05 mM/kg of each CD and AA (ranging from 0.05–0.35 M/kg)	The complex α-CD/AA in water is enthalpically stabilized, with the drug interacting stronger to α-CD than β-CD.	[142]
β-CD	AA	Carboxymethyl starch (CMS)-β-CD microgels containing AA	Increase in AA concentration led to an increase in its loading, although after 50 mg/mL it led to a decrease in encapsulation efficiency. Increasing the β-CD-CMS enhanced AA loading ratio and encapsulation efficiency.	[143]
β-CD	AA	Aqueous solutions containing AA-β-CD inclusion complexes prepared by kneading, co-precipitation and freeze-drying.	All preparation methods were successful in obtaining inclusion complexes of AA-CDs.	[144]
HP-β-CD	AA	Solutions with AA/HP-β-CD inclusion complexes prepared by solvent evaporation method, in different molar ratios.	The inclusion molar ratio was estimated to be 1:1. When higher or if the concentration of HP-β-CD increases, the drug’s properties will not be enhanced. Complexation with HP-β-CD improves the stability and bioavailability of Vitamin C. This system can be applied for unstable bioactive compounds.	[145]
HP-β-CD	AA	Solid complexes with 1:1 molar ratio of HP-β-CD-AA or 1:1:1: molar ratio of HP-β-CD-AA-triethanolamine. AA and HP-β-CD were present in 11 mg/mL and 76.4 mg/mL, respectively.	HP-β-CD can interact with AA and increase its chemical stability. Complexation stabilized the drug in acidic pH but had the opposite effect in alkaline pH. In addition, preparation of the complexes can be done in a solid state, since partial complexation of AA into HP-β-CD was observed.	[146]
α-CD, PEG-α-CD, 6-armPEG-α-CD	AA	For stability studies, 0.001% *w*/*v* of ascorbic acid solutions were used, with various CD concentrations (0.01, 0.02, 0.04, 0.08, and 0.1% *w*/*v*). For in vitro release studies, transdermal patches coated with AA, CDs (both 0.5% *w*/*w* of the Polymer Matrix), and propylene glycol (5% *w*/*w* of the Polymer Matrix) were used.	α-CD alone or complexed with Poly(ethylene glycol) enhanced the release and skin penetration of AA, with 6-armPEG-α-CD showing better results and advantages, such as low toxicity or skin irritation, adequate solubility, and peeling effects of lower concentrations of citric acid moieties.	[147]
HP-β-CD	EPC-K1	Aqueous solutions containing 0.1 mM of EPC-K1 and HP-β-CD ranging from 0.1 to 0.5 mM	Complexation with CDs inhibited the foaming generated by EPC-K1, concentration dependent, by reversing the decrease in surface tension of the solution. This system is useful for topical liquid preparations.	[148]
γ-CD	ASCP	Ternary inclusion complexes made of urea, ASCP and γ-CD by either physical mixture or ground mixture. All samples were in solid state.	Complexation with CDs, along with urea, enhanced the solubility and thermal stability of vitamin C, with the best results obtained from ground mixture at the molar ratio (1/2) of AU (ASCP/Urea = 1/12)/γ-CD	[149]
γ-CD and γ-CD-MOF	LASCP and LASCDP	Physical mixture containing 1:1 molar ratio of LASCP or LASCDP, and γ-CD-MOF, originated from aqueous solution containing 1 mM of γ-CD and KOH. Evaporated samples containing 1:1, 1:2 of ASCP with CD-MOF-1 or 1:1, 1:2, 1:4 of ASCDP with γ-CD-MOF.	γ-CD-MOF formed inclusion complexes with the lipophilic side chains of LASCP and LASCDP, at molar ratios of 1:2 for LASCP/γ-CD-MOF and 1:4 for LASCDP/γ-CD-MOF. γ-CD-MOF is useful in developing drug carriers for LASCP and LASCDP.	[150]

## Data Availability

No new data were created.

## Abbreviations

AA	Ascorbic acid
ASCP	Ascorbyl palmitate
BM-DBM	Butyl-methoxydibenzoylmethane
CD	Cyclodextrin
CDs	Cyclodextrins
Chol-βCD-Ac	Heptakis(2,3-di-O-acetyl)-hexakis-(6-o-acetyl)-6I-(cholest-5-en-3β-yloxy carbonyl)amino-6I-deoxycyclomaltoheptaose
DIMEB	Di-O-methyl-beta-cyclodextrin
Dimethyl-β-CD	Dimethyl-β-Cyclodextrin
EH-DMAB	Ethylhexyl-p-dimethylaminobenzoate
EPC-K1	L-ascorbic acid 2-[3, 4-dihydro-2,5,7,8-tetramethyl-2-(4,8,12-trimethyl-tridecyl)-2H-1-benzopyran-6-yl-hydrogen phosphate potassium salt
EPI-β-CD	Epichlorohydrin-β-Cyclodextrin
E-γ-CD	Ethyl-γ-cyclodextrin
HP-α-CD	Hydroxy-propyl-α-Cyclodextrin
HPβ-CD	Hydroxy-propyl-β-Cyclodextrin
HP-βCD-NW	Hydroxy-propyl-β-Cyclodextrin Nanofibrous webs
HP-γ-CD	Hydroxy-propyl-γ-Cyclodextrin
HP-γ-CD-NW	Hydroxy-propyl-γ-Cyclodextrin Nanofibrous webs
LAA	L-Ascorbic acid
LASCDP	L-ascorbyl 2,6-palmitate
LASCP	L-ascorbyl 6-palmitate
O-β-CD	Oleamido-β-Cyclodextrin
O-γ-CD	Octyl-γ-cyclodextrin
PBSA	Phenylbenzimidazole sulphonic acid
PEG-α-CD	Poly(ethylene glycol) citrate-α-cyclodextrin
PVRA	Vitamin A propionate
p-β-CD	β-Cyclodextrin polymer
p-β-CDC	pβ-CD cross-linked by citric acid
p-β-CDE	pβ-CD cross-linked by epichlorohydrin
pγCD	γ-Cyclodextrin polymer
RA	Retinoic acid
RAMEB	heptakis(2,6-di-O-methyl)-β-cyclodextrin
RM-β-CD	Random Methyl-β-Cyclodextrin
S-β-CD	Stearamido-β-CD
SBE-β-CD	Sulfobutylether-β-Cyclodextrin
Trans-EHMC	Trans-2-ethylhexyl-p-methoxycinnamate
VAP	Vitamin A palmitate
VC-IP	Vitamin C ascorbyl tetraisopalmitate
α-CD	α-Cyclodextrin
β-CD	β-Cyclodextrin
γ-CD	γ-Cyclodextrin
γ-CD-MOF	γ-Cyclodextrin-based metal–organic framework
2-HP-β-CD	2-hydroxypropyl-β-cyclodextrin
6-armPEG-α-CD	Six arm poly(ethylene glycol) citrate-α-cyclodextrin
